# *In silico* genome-scale metabolic modeling and *in vitro* static time-kill studies of exogenous metabolites alone and with polymyxin B against *Klebsiella pneumoniae*

**DOI:** 10.3389/fphar.2022.880352

**Published:** 2022-08-04

**Authors:** Wan Yean Chung, Nusaibah Abdul Rahim, Mohd Hafidz Mahamad Maifiah, Naveen Kumar Hawala Shivashekaregowda, Yan Zhu, Eng Hwa Wong

**Affiliations:** ^1^ School of Pharmacy, Taylor’s University, Subang Jaya, Selangor, Malaysia; ^2^ Faculty of Pharmacy, University of Malaya, Kuala Lumpur, Malaysia; ^3^ International Institute for Halal Research and Training (INHART), International Islamic University Malaysia (IIUM), Gombak, Selangor, Malaysia; ^4^ Center for Drug Discovery and Molecular Pharmacology (CDDMP), School of Pharmacy, Taylor’s University, Subang Jaya, Selangor, Malaysia; ^5^ Infection Program and Department of Microbiology, Biomedicine Discovery Institute, Monash University, Clayton, VIC, Australia; ^6^ School of Medicine, Taylor’s University, Subang Jaya, Selangor, Malaysia

**Keywords:** *Klebsiella pneumoniae*, polymyxin, metabolite, genome-scale metabolic modeling, time–kill, metabolic modulation, antimicrobial resistance

## Abstract

Multidrug-resistant (MDR) *Klebsiella pneumoniae* is a top-prioritized Gram-negative pathogen with a high incidence in hospital-acquired infections. Polymyxins have resurged as a last-line therapy to combat Gram-negative “superbugs”, including MDR *K. pneumoniae*. However, the emergence of polymyxin resistance has increasingly been reported over the past decades when used as monotherapy, and thus combination therapy with non-antibiotics (e.g., metabolites) becomes a promising approach owing to the lower risk of resistance development. Genome-scale metabolic models (GSMMs) were constructed to delineate the altered metabolism of New Delhi metallo-β-lactamase- or extended spectrum β-lactamase-producing *K. pneumoniae* strains upon addition of exogenous metabolites in media. The metabolites that caused significant metabolic perturbations were then selected to examine their adjuvant effects using *in vitro* static time–kill studies. Metabolic network simulation shows that feeding of 3-phosphoglycerate and ribose 5-phosphate would lead to enhanced central carbon metabolism, ATP demand, and energy consumption, which is converged with metabolic disruptions by polymyxin treatment. Further static time–kill studies demonstrated enhanced antimicrobial killing of 10 mM 3-phosphoglycerate (1.26 and 1.82 log_10_ CFU/ml) and 10 mM ribose 5-phosphate (0.53 and 0.91 log_10_ CFU/ml) combination with 2 mg/L polymyxin B against *K. pneumoniae* strains. Overall, exogenous metabolite feeding could possibly improve polymyxin B activity *via* metabolic modulation and hence offers an attractive approach to enhance polymyxin B efficacy. With the application of GSMM in bridging the metabolic analysis and time–kill assay, biological insights into metabolite feeding can be inferred from comparative analyses of both results. Taken together, a systematic framework has been developed to facilitate the clinical translation of antibiotic-resistant infection management.

## 1 Introduction

The emergence of multidrug-resistant (MDR) bacterial pathogens, including carbapenem-resistant *Klebsiella pneumoniae*, has garnered regular warnings of the World Health Organization (World Health Organization, 2020) and the U.S. Centers for Disease Control and Prevention (U.S. Centers for Disease Control and Prevention, 2021). Polymyxins (i.e., polymyxin B and colistin) are a group of lipopeptide antibiotics that are used as a last resort to treat severe infections caused by Gram-negative “superbugs”. Resistance can emerge during polymyxin monotherapy, which is mainly mediated by lipid A modifications in *K. pneumoniae* (Baron et al., 2016). Recently, the increasing prevalence of the mobile resistance gene *mcr* in *Enterobacterales* places critical challenges to polymyxin use (Liu et al., 2016; Yang et al., 2018; Hadjadj et al., 2019), underlining the urgent need for a novel antimicrobial therapeutic strategy. In clinics, colistin and polymyxin B are either used alone or in combination with other antimicrobials to treat life-threatening infections due to carbapenem-resistant *K. pneumoniae* (Nang et al., 2021; Yang et al., 2021)*.* The emergence of polymyxin resistance in *K. pneumoniae* clinical isolates through diverse genetic adaptation has renewed the research focus on the importance of combination therapy. Furthermore, polymyxin dosage is limited by its nephrotoxicity and neurotoxicity (Aggarwal and Dewan, 2018). Combination therapies of polymyxin antibiotics are often employed to inhibit the resistance emergence and minimize the potential toxicity (Bergen et al., 2019). Among the combination treatments, using non-antibiotic adjuvants such as exogenous metabolites together with polymyxin B is a promising approach as the use of metabolites at low concentrations is generally non-toxic to the host (Chengxue et al., 2014; Zeng et al., 2017; Jiang et al., 2020; Rosenberg, Fang and Allison, 2020; Wang et al., 2020).

Recent studies have demonstrated that the cellular metabolism of bacterial pathogens is critical for antimicrobial efficacy (Liu et al., 2019). Modulation of cellular metabolism *via* exogenous metabolite feeding could significantly elevate antibiotic susceptibility of drug-resistant bacteria (Zeng et al., 2017; Su et al., 2018; Yang et al., 2019). However, the complicated interplay of multiple metabolic pathways underlying the synergy of metabolite–antimicrobial combination remains unclear, thus hampering the discovery of effective metabolite adjuvants to improve antimicrobial efficacy, including the last-line polymyxins. A genome-scale metabolic model (GSMM) serves as a systematic tool to simulate metabolic flux changes in response to antimicrobial treatment and metabolite feeding (Wadhwa et al., 2018; Rizvi et al., 2019; Zhou et al., 2021), and thus it can assist in delineating the mechanisms of enhanced bacterial killing by exogenous metabolite feeding.

The primary aim of this study was to identify promising polymyxin B–metabolite combinations against MDR *K. pneumoniae* using GSMM coupled with time–kill studies. Four GSMMs were constructed to elucidate the metabolic adaptation of *K. pneumoniae* strains upon addition of metabolites. We reveal that rewiring of metabolic flux distribution occurred owing to the feeding of additional metabolites. We also show that increased antimicrobial activity was demonstrated by the combination of 3-phosphoglycerate (3PG) and ribose 5-phosphate (R5P) with polymyxin B against New Delhi metallo-β-lactamase (NDM)- and extended spectrum β-lactamase (ESBL)-producing isolates.

## 2 Materials and methods

### 2.1 Bacterial isolates

Four *K. pneumoniae* American Type Culture Collection (ATCC) isolates were analyzed: ATCC 10031, 700603 (ST489, Pasteur scheme, same for following strains), 700721 (ST38, also known as *K. pneumoniae* MGH78578), and BAA-2146 (ST11). The strains were selected to represent a mixture of strains susceptible and resistant to polymyxin B (Table 1) and MDR strains. Strain ATCC 700603 was originally isolated from a urine sample of a hospitalized patient in 1994 (Elliott et al., 2016) and produces multiple ESBLs, especially beta-lactamase SHV-18. Strain ATCC BAA-2146 is an NDM-producing reference strain. All strains were purchased from ATCC and were stored in tryptone soy broth with 20% glycerol at −80°C.

**TABLE 1:** MICs of *K. pneumoniae* isolates.

*K. pneumoniae* isolate	Polymyxin B MIC (mg/L)
ATCC 10031	4
ATCC 700603	2
ATCC 700721	2
ATCC BAA-2146	2

### 2.2 Genome-scale metabolic modeling

The draft models were initially constructed by CarveMe (Machado et al., 2018) using genome annotation and coded in System Biology Markup Language Level 3 Version 1 (Smith et al., 2010). Manual curation and metabolic simulations were performed using COBRApy (Ebrahim et al., 2013). Transport and exchange reactions were added to allow nutrient uptake and metabolite transport across membranes according to the BiGG database (Norsigian et al., 2020). The manually added metabolites were complemented with specific properties including compartment localization, charge, formula, name, and database identifier according to the BiGG database.

For simulation of bacterial growth in minimal media (M9), the maximum uptake rates of nutrient ingredients were set to 10 mmol·gDW^−1^·h^−1^ (Zhu et al., 2018) whereas for Mueller–Hinton (MH) medium, the maximum uptake rates of nutrient ingredients were empirically constrained to 1 mmol·gDW^−1^·h^−1^ (Zhu et al., 2019). Non-growth-associated maintenance ATP consumption was set to 10 mmol·gDW^−1^·h^−1^ according to the previous study (Zhu et al., 2018).

Seven exogenous metabolites tested in this study are phenylpyruvate (PHPYR), orotate (OROT), 3-phosphohydroxypyruvate (3PHP), glycerol 3-phosphate (GLYC3P), 3PG, R5P, and uridine 5ʹ-diphospho-*N*-acetylglucosamine (UACGAM). The MH medium was used for metabolic modeling. For each metabolite, additional transport reactions were incorporated into the draft model, and the maximum uptake rate was constrained to 10 mmol·gDW^−1^·h^−1^. The metabolic solution space was sampled with 10,000 random points using OptGpSampler (Megchelenbrink, Huynen and Marchiori, 2014). Flux distributions of metabolite feeding were then compared with those of non-feeding conditions.

### 2.3 Antibiotic and exogenous metabolites

Polymyxin B was purchased from Merck (Darmstadt, Hesse) and was prepared by dissolving with Milli-Q water to obtain a final concentration of 512 mg/L. The exogenous metabolites (10 mM PHPYR, 1 mM OROT, 5 mM 3PHP, 10 mM 3PG, 10 mM R5P, and 1 mM UACGAM) were individually examined, alone and in combination with 2 mg/L polymyxin B against the four *K. pneumoniae* strains by static time–kill studies. The concentrations of exogenous metabolites were normalized to deliver 60 mM carbon except OROT, 3PHP, and 3PG due to their poor aqueous solubility. All metabolites were purchased from Sigma-Aldrich (Saint Louis, Missouri).

### 2.4 Static time–kill studies

Static time–kill studies were conducted over 24 h to study antimicrobial activity and the emergence of resistance after treatment with polymyxin B (Lin et al., 2019; Wistrand-Yuen et al., 2020). *K. pneumoniae* isolates were investigated at an initial inoculum of 10^6^ CFU/ml [standard inoculum, as per the Clinical and Laboratory Standards Institute (CLSI) guidelines]. Log-phase cultures of *K. pneumoniae* isolates were prepared prior to the experiments.

Before spiking in antimicrobial agents, a sample of *t* = 0 h was collected. Clinically relevant free unbound concentration of polymyxin B 2 mg/L was used. After spiking in antimicrobial agents, further samples (∼700 μl) at *t* = 1, 4, and 24 h were collected aseptically, diluted appropriately in 0.9% saline solution, and plated manually. Upon incubation at 35°C for 24 h, viable cell counting was conducted. The final cell viability was expressed in log_10_ CFU/ml.

Polymyxin B exerted rapid bactericidal activity within 1 h, but significant bacterial regrowth was observed following 24 h exposure to polymyxin B monotherapy (Lin et al., 2019). Hence, the pharmacodynamic effect of the combination treatment was assessed over 24 h to investigate bacterial regrowth. Findings from polymyxin B pharmacokinetic studies suggest that the currently recommended mean polymyxin B maximum serum concentration at steady-state ranges from ∼2 to 14 mcg/ml (Avedissian et al., 2019). The polymyxin B concentrations selected were based on the clinical dosing regimens (Tsuji et al., 2019).

### 2.5 Pharmacodynamic analysis

Pharmacodynamic analysis was carried out to determine microbiological response to antimicrobial treatment (Lin et al., 2019). The log change method (log change = [log_10_ (CFU_t_)−log_10_ (CFU_0_)]) was used, comparing the change in bacterial count from 0 h to time point of interest. For static time–kill studies, antibacterial activity involves a reduction of ≥1 log_10_ CFU/ml from the initial inoculum. Bactericidal activity was defined as ≥ 3 log_10_ CFU/ml reduction from the starting inoculum. Additivity and synergy were defined as 1.0 to <2 log_10_ CFU/ml and ≥2 log_10_ CFU/ml reduction with the combination relative to its most active single agent, respectively (Sharma et al., 2017). Antagonism was defined as a ≥1 log_10_ CFU/ml increase between the combination and the most active single agent (Shields et al., 2018; Barber et al., 2021).

## 3 Results

### 3.1 Construction of genome-scale metabolic models for selected *K. pneumoniae* strains

With the aim of identifying promising metabolite adjuvants to increase antimicrobial activity of polymyxin B against *K. pneumoniae*, we have studied polymyxin-resistant strain ATCC 10031, polymyxin-susceptible strain ATCC 700721, and polymyxin-susceptible but MDR *K. pneumoniae* strains (ATCC 700603 and BAA-2146) (Table 1 **)**. In addition, GSMMs were constructed to simulate flux changes upon metabolite addition. Initial draft models were developed for the four *K. pneumoniae* isolates based on genome annotation. During manual curation against the literature and databases, a total of 10–12 metabolites and 20–23 reactions were added to each model **(** Supplementary Table S1 **)**, enabling metabolite uptake and secretion. The resulting models were designated iKpne_ATCC10031_21 (ATCC 10031), iKpne_ATCC700603_21 (ATCC 700603), iKpne_ATCCBAA2146_21 (ATCC BAA-2146), and iKpne_ATCC700721_21 (ATCC 700721) according to naming convention, and each of them contains 2,531–2,713 reactions, 1,695–1,778 metabolites, and 1,292–1,612 genes (Table 2).

**TABLE 2:** Total number of genes, metabolites, and reactions in the constructed GSMMs.

GSMM	Gene	Metabolite	Reaction
iKpne_ATCC10031_21	1,292	1,703	2,531
iKpne_ATCC700603_21	1,612	1,778	2,708
iKpne_ATCC700721_21	1,587	1,778	2,713
iKpne_ATCCBAA2146_21	1,572	1,695	2,611

### 3.2 Genome-scale metabolic modeling

The four models predicted the maximum specific growth rate (*μ*_max_) of 0.92 and 1.05 h^−1^ in M9 and MH media, respectively. The predicted *μ*_max_ in MH media is similar to the calculated *μ*_max_ using time–kill data, which varied between 1.02–1.16 h^−1^ for the *K. pneumoniae* isolates.

The metabolites were selected based on the previous transcriptomic and metabolomic findings (Maifiah et al., 2017; Han et al., 2018; Hussein et al., 2018; Abdul Rahim et al., 2021), which indicated that the intracellular levels of metabolites R5P, UACGAM, and GLYC3P were significantly perturbed by polymyxin. Furthermore, metabolites PHPYR, OROT, 3PG, and 3PHP have also been identified as significant metabolites perturbed by the combination (Abdul Rahim et al., 2021). Although many significant metabolites were identified from the studies, the selected metabolites were those that demonstrated perturbations to both gene expression and metabolism of the same pathway [e.g., *gnd* and R5P in the pentose phosphate pathway (PPP); *pgk* and 3PG in gluconeogenesis] by the combination (Abdul Rahim et al., 2020; 2021). For instance, transcriptomics and metabolomic results revealed that the expression of gene *gnd* and abundance level of R5P were downregulated and decreased in response to the polymyxin combination treatment, respectively. Thus, these observations were believed to further strengthen the basis of selection.

**FIGURE 1:**
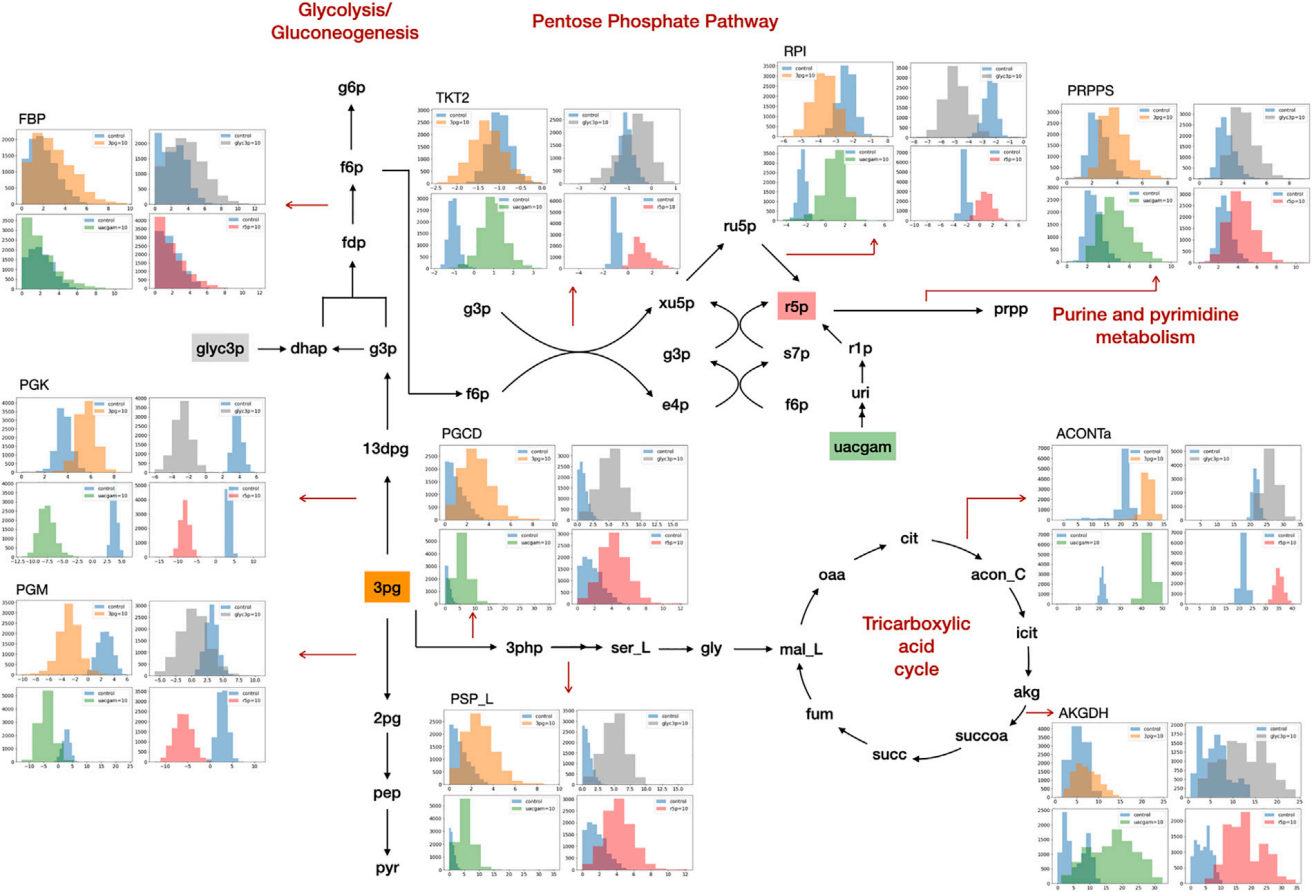
Metabolite feeding of 3PG, GLYC3P, R5P, and UACGAM induced metabolic alterations. The subgraphs indicate the distribution of sampled metabolic fluxes (mmol·gDW^−1^·h^−1^) in iKpne_ATCC700603_21 (blue, control; orange, 3PG; gray, GLYC3P; red, R5P; green, UACGAM). The metabolite abbreviations are as follows: g6p, D-glucose 6-phosphate; f6p, D-fructose 6-phosphte; fdp, D-fructose 1,6-biphosphate; dhap, dihydroxyacetone phosphate; g3p, glyceraldehyde 3-phosphate; 13dpg, 3-phospho-D-glyceroyl phosphate; 3pg, 3-phosphoglycerate; 2pg, D-glycerate 2-phosphate; pep, phosphoenolpyruvate; pyr, pyruvate; ru5p, D-ribulose 5-phosphate; xu5p, D-xylulose 5-phosphate; r5p, D-ribose 5-phosphate; s7p, sedoheptulose 7-phosphate; e4p, D-erythrose 4-phosphate; prpp, 5- phospho-alpha-D-ribose 1-diphosphate; 3php, 3-phosphohydroxypyruvate; ser_L, L-serine; gly, glycine; mal_L, L-malate; oaa, oxaloacetate; cit, citrate; acon_C, cis-aconitate; icit, isocitrate; akg, 2-oxoglutarate; succoa, succinyl-CoA; succ, succinate; fum, fumarate. The reaction abbreviations are as follows: FBP, fructose-bisphosphatase; PGK, phosphoglycerate kinase; PGM, phosphoglycerate mutase; TKT2, transketolase 2; RPI, ribose-5-phosphate isomerase; PRPPS, phosphoribosylpyrophosphate synthetase; PGCD, phosphoglycerate dehydrogenase; PSP_L, phosphoserine phosphatase; ACONTa, aconitase (half-reaction A); AKGDH, 2-oxoglutarate dehydrogenase.

GSMM simulation results show that the addition of PHPYR, OROT, and 3PHP resulted in limited impact on non-central metabolic pathways, whereas feeding of 3PG, GLYC3P, R5P, and UACGAM induced significant metabolic perturbations to multiple pathways, including central metabolism (Figure 1). GLYC3P was excluded for further analyses due to its similar impact as 3PG. The perturbed reaction specific flux values under control and metabolite feeding treatment are denoted in the format **flux**_**control**_ **/flux**_**metabolite**_ in brackets in Sections 3.2.1, 3.2.2.

#### 3.2.1 Metabolic impact on non-central metabolism

The model simulations predict that the uptake of exogenous PHPYR was at a relatively low rate compared to that of other metabolites and exerted minimal effect on phenylalanine metabolism upon feeding. Generally, GSMM results show that the addition of OROT would increase pyrimidine biosynthesis. A higher flux distribution of orotate phosphoribosyltransferase (ORPT) (iKpne_ATCC10031_21: 0.41/0.54; iKpne_ATCC700603_21: 0.20/0.43; iKpne_ATCC700721_21: 0.22/0.48; and iKpne_ATCCBAA2146_21: 0.19/0.42), orotidine 5′-phosphate decarboxylase (OMPDC) (iKpne_ATCC10031_21: 0.41/0.54; iKpne_ATCC700603_21: 0.20/0.43; iKpne_ATCC700721_21: 0.22/0.48; and iKpne_ATCCBAA2146_21:0.19/0.42), and uridine 5′-monophosphate kinase (UMPK) (iKpne_ATCC10031_21: 1.67/1.76; iKpne_ATCC700603_21: 2.64/2.87; iKpne_ATCC700721_21: 2.57/2.58; and iKpne_ATCCBAA2146_21: 2.59/2.65) indicated elevated pyrimidine biosynthesis activity. Uridine diphosphate (UDP) was further converted to uridine-5′-triphosphate (UTP) via higher flux through nucleoside-diphosphate kinase (NDPK2) (iKpne_ATCC10031_21: 5.99/6.17; iKpne_ATCC700603_21: 4.92/5.20; iKpne_ATCC700721_21: 4.65/4.55; and iKpne_ATCCBAA2146_21: 5.18/5.14). Moreover, the addition of exogenous 3PHP was predicted to digest into serine and glycine metabolism to increase fluxes of phosphoserine transaminase (PSERT) (iKpne_ATCC10031_21: 2.29/11.43; iKpne_ATCC700603_21: 0.89/10.67; iKpne_ATCC700721_21: 1.47/10.62; and iKpne_ATCCBAA2146_21: 1.29/10.57), phosphoserine phosphatase (PSP_L) (iKpne_ATCC10031_21: 2.29/11.43; iKpne_ATCC700603_21: 0.89/10.67; iKpne_ATCC700721_21: 1.47/10.62; and iKpne_ATCCBAA2146_21: 1.29/10.57), and then glycine hydroxymethyltransferase (GHMT2r) (iKpne_ATCC10031_21: 1.11/6.00; iKpne_ATCC700603_21: 2.15/4.33; iKpne_ATCC700721_21: 1.27/4.98; and iKpne_ATCCBAA2146_21: 0.90/6.04) to form glycine.

#### 3.2.2 Metabolic impact on central metabolism

**FIGURE 2:**
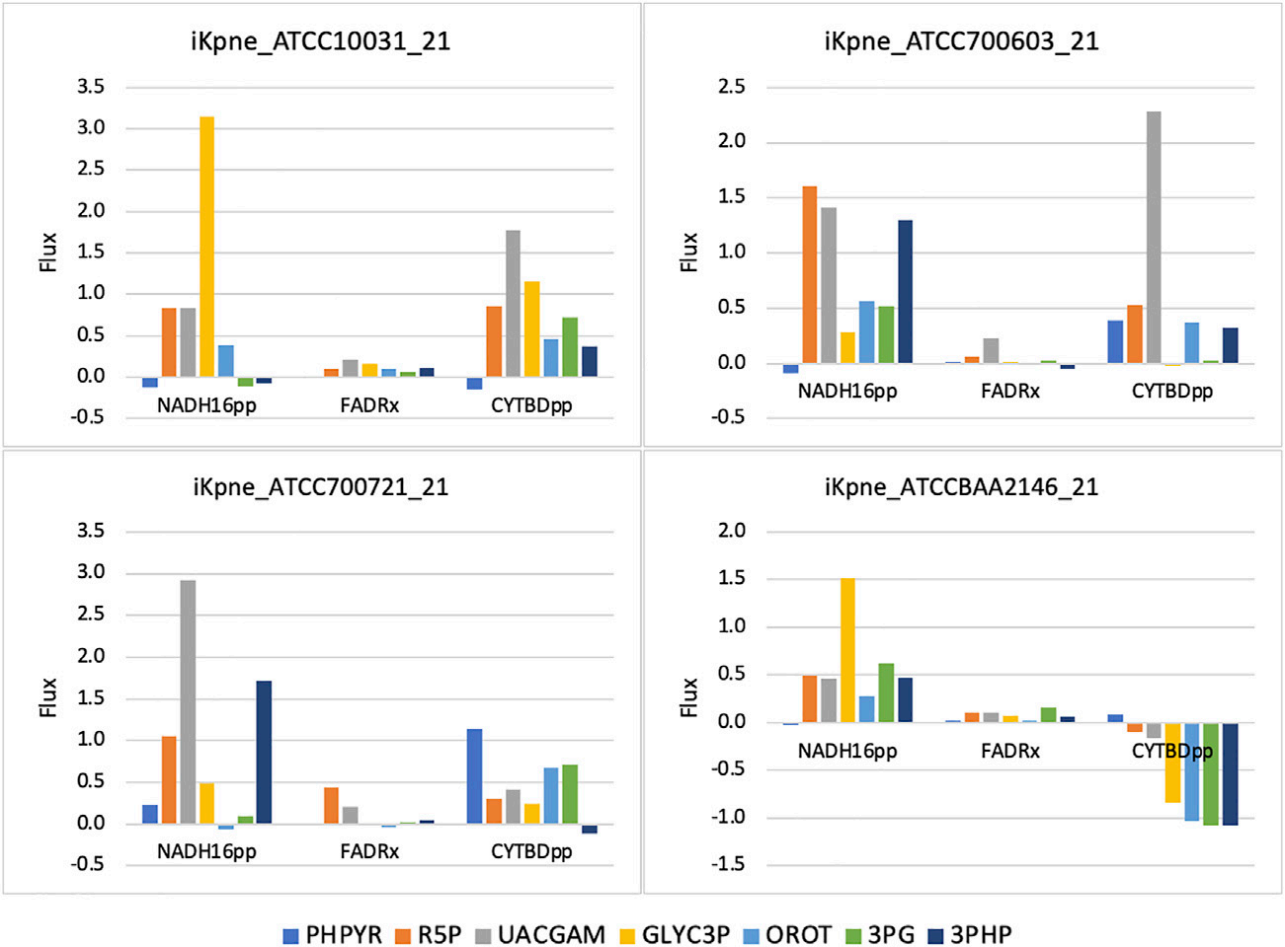
Oxidative phosphorylation fluxes changes upon metabolite addition. The reaction abbreviations are as follows: NADH16pp, NADH dehydrogenase (ubiquinone-8 and 3 protons) (periplasm); FADRx, FAD reductase; CYTBDpp, cytochrome oxidase bd (ubiquinol-8: 2 protons) (periplasm).

GSMM results show that feeding of 3PG resulted in increased glycolytic/gluconeogenetic fluxes in all four strains (Figure 1). Results show that 3PG influx bifurcates to form D-glycerate 2-phosphate (2PG) of glycolysis and 3-phospho-D-glyceroyl phosphate (13DPG) of gluconeogenesis; the latter in turn enhances PPP flux to generate R5P. Results show enhanced production of 5-phospho-alpha-D-ribose 1-diphosphate (PRPP), the starting metabolite of the nucleotide biosynthesis pathway (Figure 1) and increased fluxes of reactions ORPT, OMPDC, UMPK, and NDPK2 toward UTP biosynthesis. Furthermore, addition of 3PG was predicted to increase serine biosynthesis *via* enhanced fluxes of PSERT (iKpne_ATCC10031_21: 2.29/5.39; iKpne_ATCC700603_21: 0.89/3.70; iKpne_ATCC700721_21: 1.47/3.85; and iKpne_ATCCBAA2146_21: 1.29/4.43) and PSP_L (iKpne_ATCC10031_21: 2.29/5.39; iKpne_ATCC700603_21: 0.89/3.70; iKpne_ATCC700721_21: 1.47/3.85; and iKpne_ATCCBAA2146_21: 1.29/4.43). Increased tricarboxylic acid cycle (TCA) cycle flux was observed upon feeding of 3PG. Additionally, the overall fluxes within oxidative phosphorylation were increased (Figure 2) which potentially resulted in higher oxygen consumption and a higher ATP turnover rate.

Furthermore, the GSMM predicted the exogenous GLYC3P formed dihydroxyacetone phosphate (DHAP) through enhanced dehydrogenation (iKpne_ATCC10031_21: 4.15/4.26; iKpne_ATCC700603_21: −4.92/1.80; iKpne_ATCC700721_21: −3.92/3.95; and iKpne_ATCCBAA2146_21: −4.15/3.06), which in turn flew down to glycolysis, serine metabolism, and eventually to the TCA cycle (Figure 1). The metabolic flux changes caused by GLYC3P feeding are similar to 3PG feeding. In addition, feeding of R5P was predicted to significantly affect central carbon metabolism flux. The addition of R5P would preferably form D-ribulose 5-phosphate (RU5P) than PRPP via isomerization (iKpne_ATCC10031_21: −0.44/5.80; iKpne_ATCC700603_21: −2.42/1.48; iKpne_ATCC700721_21: −2.59/0.54; and iKpne_ATCCBAA2146_21: 2.56/1.19). Increased flux of generating fructose 6-phosphate (F6P) from RU5P would enter glycolysis metabolism, and then the end product of glycolysis, acetyl CoA, would be fueled to the TCA cycle for cellular respiration. Furthermore, the GSMM results also reveal that feeding of UACGAM increases the fluxes of central and nucleotide metabolism. The exogenous UACGAM flows into PPP through the nucleotide salvage pathway (Figure 1) *via* increased flux of pyrimidine-nucleoside phosphorylase (iKpne_ATCC700603_21: −1.42/6.54; iKpne_ATCC700721_21: −1.32/6.40; and iKpne_ATCCBAA2146: −1.00/6.91) except for iKpne_ATCC10031_21. Model iKpne_ATCC10031 predicted exogenous UACGAM digested into PPP via increased flux of uridine hydrolase (URIH) (iKpne_ATCC10031_21: 0.44/9.85).

### 3.3 Validation of metabolite effects using *in vitro* time–kill studies

**FIGURE 3:**
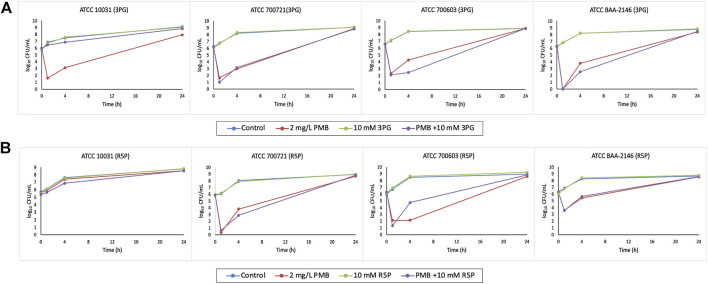
Time–kill curves of metabolite treatment with polymyxin B (PMB), alone and in combination. **(A)** 10 mM 3PG and **(B)** 10 mM R5P.

Polymyxin B (2 mg/L) monotherapy produced rapid and extensive killing within 1 h against all isolates except ATCC 10031 with ≥3 log_10_ CFU/ml killing (Figures 3A,B). Nevertheless, significant bacterial regrowth was observed at 24 h for all isolates treated with polymyxin B monotherapy.

For the six metabolites tested, three metabolite–polymyxin B combinations demonstrated enhanced antimicrobial activity against MDR *K. pneumoniae* isolates even when NDM was present.

The combination of polymyxin B (2 mg/L) with 10 mM 3PG resulted in strong bacterial killing at 1 h with 4.3–6.2 log_10_ CFU/ml reduction for isolate ATCC 700603 and BAA-2146 compared to initial inoculum (Figure 3A). At 4 h, the combination treatment increased the extent of antibacterial activity approximately to 2 log_10_ CFU/ml (1.82 log_10_ CFU/ml) reduction for isolate ATCC 700603 relative to its most active polymyxin B monotherapy (Figure 3A). A similar increased antibacterial effect was also observed for the combination treatment against MDR isolate BAA-2146 with 1.26 log_10_ CFU/ml reduction at 4 h (Figure 3A). However, bacterial regrowth was observed for both isolates at 24 h.

Metabolite feeding with 10 mM R5P combined with polymyxin B showed a bacterial count reduction of approximately 1 log_10_ CFU/ml (0.91 log_10_ CFU/ml) for isolate ATCC 700721 (Figure 3B). Interestingly, isolate ATCC 10031 is resistant to polymyxin B monotherapy and the addition of R5P to polymyxin B resulted in a modest improvement in antibacterial activity with 0.53 log_10_ CFU/ml reduction compared with polymyxin B monotherapy at 4 h (Figure 3B).

The antibacterial effect of UACGAM feeding was also tested against MDR isolates. For ESBL isolate ATCC 700603, addition of 1 mM UACGAM to polymyxin B treatment showed an increase of bacterial killing of 1 log_10_ CFU/ml reduction. The magnitude of antibacterial activity was further enhanced to 0.70 log_10_ CFU/ml reduction at 4 h in contrast to polymyxin B monotherapy (Supplementary Figure S1).

## 4 Discussion

The rapid spread of opportunistic *K. pneumoniae* that are resistant to last-resort polymyxins highlights the urgent requirement for novel antimicrobial adjuvant therapy to minimize the emergence of resistance. Polymyxin B combined with non-antibiotics, such as metabolites, offers an attractive approach to increase antibacterial activity without exceeding the clinically achieved concentration of polymyxin B. To this end, it is crucial to understand the reciprocal relationship between bacterial metabolic responses to exogenous metabolites and antimicrobial activity to optimize the combination therapy. GSMM is a powerful tool in studying bacterial metabolism, and it has been applied to elucidate the mechanism of antibiotic killing and development of resistance. Thus, integration with *in vitro* experiments enables a systematic framework for identifying novel exogenous metabolite–antibiotic combinations.

Simulation with the four GSMMs showed that additions of exogenous metabolites such as 3PG, 3PHP, GLYC3P, R5P, and UACGAM display an effect on increasing bacterial growth except for metabolites PHPYR and OROT. This could be explained by the flow of metabolic flux corresponding to the metabolite addition in which metabolite PHPYR was not digested in the metabolism; OROT addition only exerted minor effects on purine and pyrimidine metabolism. The highest growth induced by UACGAM feeding among the metabolites demonstrated the highest metabolic flux changes in model predictions. The uridine part of UACGAM can be digested to form nucleotides, whereas the amino sugar component (i.e., *N*-acetylglucosamine) can be utilized for cell envelope biosynthesis.

The growth rate is the primary variable that determines the phenotype of susceptibility to antibiotics of the bacterial populations (Martínez and Rojo, 2011). A slow growth rate was associated with low antibiotic activity (Yang, Bening and Collins, 2017; Zampieri et al., 2017; Lee et al., 2018). Thus, we hypothesize that the stagnant bacterial growth upon feeding of PHPYR and OROT would not exert antibacterial activity when treated together with polymyxin B against *K. pneumoniae* isolates. The time–kill studies supported this hypothesis where both combination therapies (i.e., polymyxin B with PHPYR; polymyxin B with OROT) did not show effect on antibacterial activity. In addition, minor metabolic flux changes in glucose metabolism and oxidative phosphorylation displayed by feeding of these two metabolites suggest that no metabolic regulation and modulation occur.

Prax et al. (2016) showed that glucose potentiated a membrane-active antimicrobial peptide, daptomycin, in which killing may be dependent on glucose metabolism. The attenuation of carbon catabolism associated with cellular respiration is the primary cause of metabolite-driven ciprofloxacin activity (Gutierrez et al., 2017). Recent metabolomics results showed polymyxin treatment-induced dramatic changes in central carbon metabolism in polymyxin-susceptible Gram-negative pathogens (Maifiah et al., 2017; Zhu et al., 2019). Our fluxomic data revealed that metabolite feeding of 3PG, R5P, UACGAM, and GLYC3P notably increased glycolysis, PPP, and TCA cycle fluxes. It is conceivable that exogenous metabolite feeding would further intensify the metabolic burden attributed to polymyxin B activity and cause increased cellular respiration. On top of that, polymyxin treatment also induced disruption of nucleotide biosynthesis (Zhu et al., 2018). *In silico* addition of the aforementioned four metabolites also upregulated purine and pyrimidine metabolism. Our time–kill result showed enhanced antimicrobial killing by the combination of 3PG, R5P, and UACGAM treated along with polymyxin B against *K. pneumoniae* (Figures 3A,B; Supplementary Figure S1). These results indicate that the surge of ATP is required to restore the disrupted nucleotide pool because of both antibiotic and metabolite treatments (Yang et al., 2019). The enhanced ATP demand stimulates the nucleotide biosynthesis metabolism and elevates the central carbon metabolism. The increased metabolic activity by metabolite feeding is likely to produce toxic metabolic by-products that reduce bacterial fitness (Stokes et al., 2019), hence increasing the killing effect of polymyxin B.

Another possible mechanism of metabolite feeding is increased production of reactive oxygen species (ROS) to enhance antibiotic activity (Brynildsen et al., 2013; Van Acker and Coenye, 2017). Increasing ROS production would increase bacterial sensitivity to oxidative attack (Brynildsen et al., 2013). The mechanism of polymyxin action involves free radical-induced death (Trimble et al., 2016). Abdul Rahim et al. (2021) postulated that an increase in nucleotide synthesis including R5P and OROT was an initial bacterial stress response to polymyxin combination treatment (Abdul Rahim et al., 2021). Such metabolic perturbation might be exacerbated driven by TCA activity upon metabolite feeding. Our results showed that metabolite feeding upregulated the TCA cycle and produced NADH which is utilized for facilitating the electron transport chain. This would induce the formation of ROS and cause oxidative damage, contributing to lethality. Altogether, the increased fluxes of NADH16pp, FADRx, and CYTBDpp (Figure 2), inducing an oxidative stress and concurrently increasing metabolic activity by metabolite feeding, may sensitize *K. pneumoniae* to polymyxin B killing.

For polymyxin B-resistant isolate ATCC 10031, evident in time–kill studies, the addition of metabolite R5P to polymyxin B resulted in a slight improvement (log change = 0.52 log_10_ CFU/ml) in antibacterial activity at 4 h compared with polymyxin B monotherapy (Figure 3B). This suggests that metabolite feeding may be a possible approach to restore antibiotic susceptibility of antibiotic-resistant isolates. Antibiotic-resistant strains are generally demonstrated to have weaker bacterial fitness and reduced metabolism due to evolution of mutation under selection pressure of antibiotics (Lázár et al., 2014). The addition of exogenous metabolites to restore the metabolic deprivation offers a hopeful approach to increase sensitivity to antibiotics of antibiotic-resistant bacteria (Cheng et al., 2019; Li et al., 2020). This enables better antimicrobial activity to be achieved with combinations containing clinically relevant polymyxin B concentrations given that polymyxin B-induced nephrotoxicity is a dose-limiting adverse effect (Avedissian et al., 2019).

Despite the positive antimicrobial effect of the combination treatment, an antagonistic effect was observed for the combination of 3PG and R5P with polymyxin B against ATCC 10031 and ATCC 700603, respectively (Figures 3A,B). Although the underlying mechanisms of these antagonism pairs remain unclear, they could be considered a potential target for the development of new antimicrobial therapy for these *K. pneumoniae* isolates. Alteration of related metabolic processes could thereby lead to a reversal of the antagonistic effect, thus improving the susceptibility of antibiotics. It would be interesting to investigate the metabolic perturbations in gene expression and metabolism in the *K. pneumoniae* isolates driven by the combination.

In summary, this is the first study incorporating GSMM findings to unveil mechanistic insights into metabolic flux changes following metabolite addition, correlated with antibiotic activity through *in vitro* studies. This will shed light on antimicrobial development of non-antibiotic combinations with polymyxin B to rescue the last-line resort. Further studies into transcriptomics and metabolomics analysis to delineate the complex metabolic responses to metabolite feeding are warranted for better model validation and accuracy. Apart from that, *in vivo* studies are crucial to evaluate the efficacy, concentration, and safety of metabolite adjuvants used in potentiating antibiotic activity against MDR *K. pneumoniae* infections.

## Data Availability

The original contributions presented in the study are included in the article/Supplementary Material; further inquiries can be directed to the corresponding authors.
