# Supplementary figures and images for: In silico genome-scale metabolic modeling and in vitro static time-kill studies of exogenous metabolites alone and with polymyxin B against Klebsiella pneumoniae
Source: Front Pharmacol. 2022 Aug 4;13:880352. doi: 10.3389/fphar.2022.880352 (PMC9386545; doi:10.3389/fphar.2022.880352)

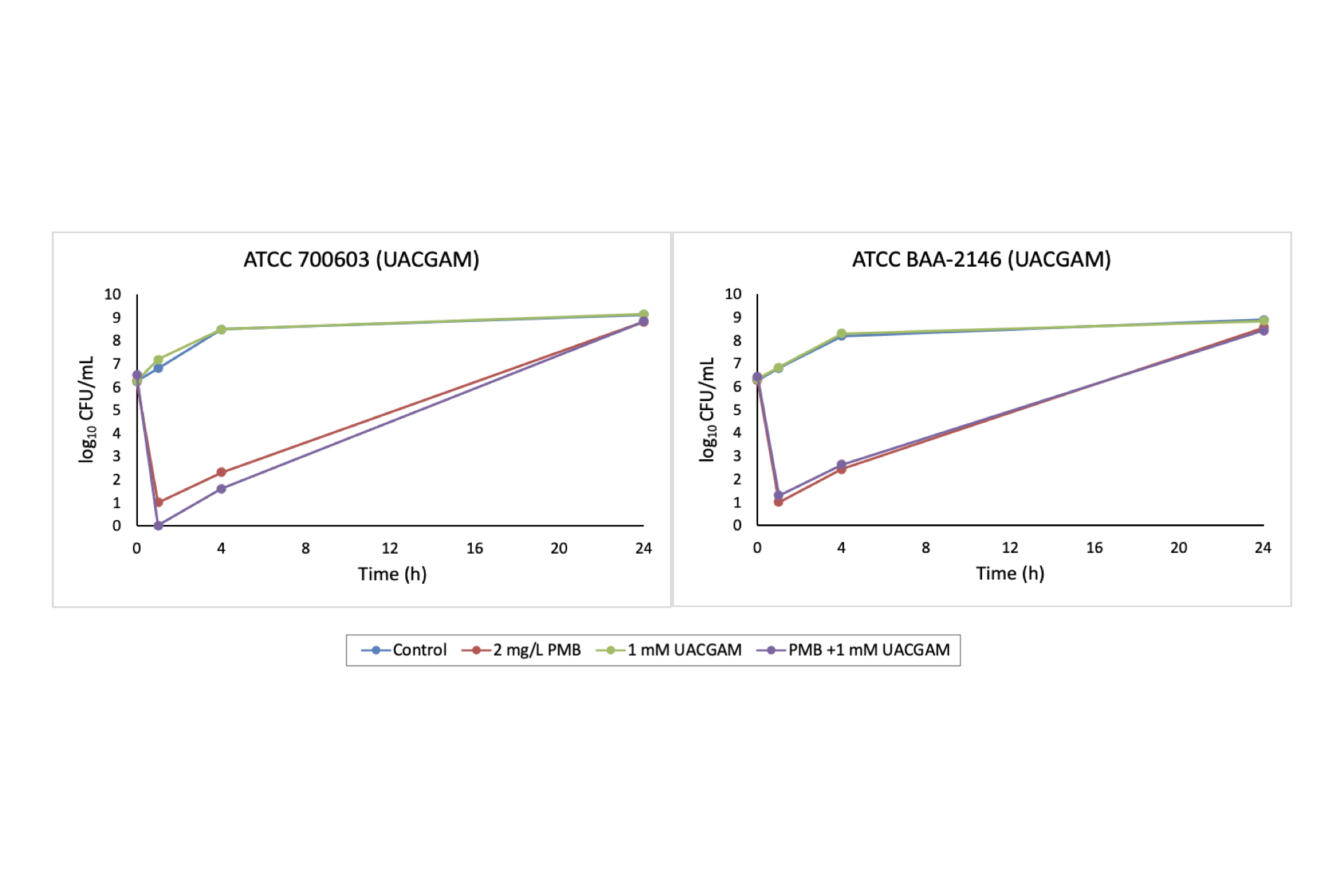

Supplement: Supplementary file 1 [file Image1.TIFF]
