# Supplementary material for: In silico genome-scale metabolic modeling and in vitro static time-kill studies of exogenous metabolites alone and with polymyxin B against Klebsiella pneumoniae
Source: Front Pharmacol. 2022 Aug 4;13:880352. doi: 10.3389/fphar.2022.880352 (PMC9386545; doi:10.3389/fphar.2022.880352)
Supplement: Supplementary file 2 [file Table1.DOCX]

**Table S1:** List of missing metabolites and reactions manually added to the GSMMs.

| **GSMM** | **Metabolite** | | **Reaction** |
| --- | --- | --- | --- |
|  | **Extracellular (e)** | **Periplasm (p)** |  |
| iKpne_ATCC10031_21  iKpne_ATCC700603_21  iKpne_ATCC700721_21  iKpne_ATCCBAA2146_21 | pydx_e | pydx_p | EX_pydx_e  PYDXtex  PYDXt2rpp |
|  | ribflv_e | ribflv_p | EX_ribflv_e  RIBFLVtex  RIBFLVt2rpp |
|  | glu__D_e | glu__D_p | EX_glu__D_e  GLUDtex  GLUDt2rpp |
|  | phpyr_e | phpyr_p | EX_phpyr_e  PHPYRtex  PHPYRt2rpp |
|  | 3php_e | 3php_p | EX_3php_e  3PHPtex  3PHPt2rpp |
|  | - | - | EX_cro4_e  EX_hg2_e  EX_na1_e  EX_ni2_e  EX_cd2_e |
| iKpne_ATCCBAA2146_21 | 3pg_e | 3pg_p | EX_3pg_e  3PGtex  3PGt2rpp |
